# Effect of Environmental pH on the Mechanics of Chitin and Chitosan: A Single-Molecule Study

**DOI:** 10.3390/polym16070995

**Published:** 2024-04-05

**Authors:** Song Zhang, Yunxu Ji, Yiwei He, Juan Dong, Haohang Li, Shirui Yu

**Affiliations:** Department of Food Science and Engineering, Moutai Institute, Renhuai 564502, China; jiyunxu@mtxy.edu.cn (Y.J.); 18185657179@139.com (Y.H.); 18586724865@163.com (J.D.); lyz20180810lyz@163.com (H.L.)

**Keywords:** chitin, chitosan, biomacromolecules, mechanical properties

## Abstract

Chitin and chitosan are important structural macromolecules for most fungi and marine crustaceans. The functions and application areas of the two molecules are also adjacent beyond their similar molecular structure, such as tissue engineering and food safety where solution systems are involved. However, the elasticities of chitin and chitosan in solution lack comparison at the molecular level. In this study, the single-molecule elasticities of chitin and chitosan in different solutions are investigated via atomic force microscope (AFM) based single-molecule spectroscopy (SMFS). The results manifest that the two macromolecules share the similar inherent elasticity in DOSM due to their same chain backbone. However, obvious elastic deviations can be observed in aqueous conditions. Especially, a lower pH value (acid environment) is helpful to increase the elasticity of both chitin and chitosan. On the contrary, the tendency of elastic variation of chitin and chitosan in a larger pH value (alkaline environment) shows obvious diversity, which is mainly determined by the side groups. This basic study may produce enlightenment for the design of intelligent chitin and chitosan food packaging and biomedical materials.

## 1. Introduction

**Figure 1:**
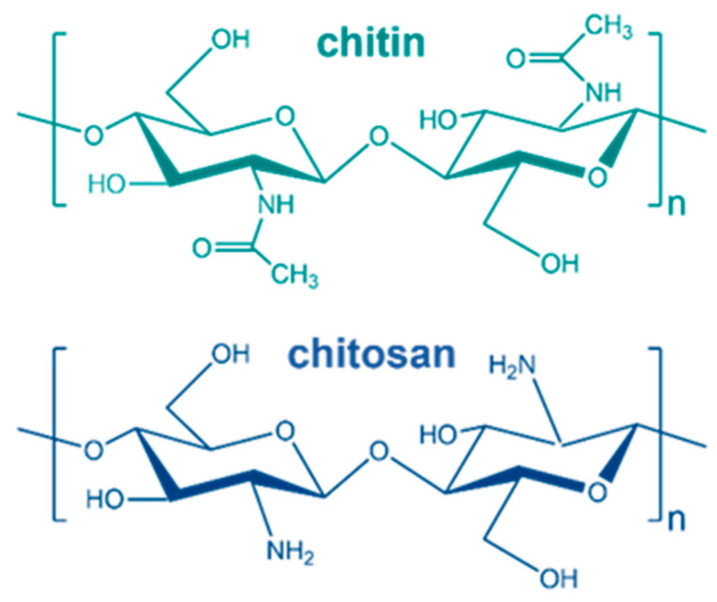
Chemical structures of chitin and chitosan.

Polysaccharides are a sort of biomacromolecule that the backbone linked by saccharide rings always serves as structural or energy storage materials [1]. Among the polysaccharides, chitin and chitosan are widely distributed in most fungus and marine animals, which are important to protect these species from the attack of external environments [2,3]. The total weight of chitin and chitosan in the cell wall of yeast can reach about 20% of the cell wall weight, which plays a key role in activities such as cell metabolism and proliferation of yeast [4]. In addition, the good crystallization properties and plentiful crystal structures of chitin and chitosan play a key role in maintaining the stability of body shape of yeast [5]. The molecular structures of chitin and chitosan are quite similar (Figure 1) since the glucose ring is the basic unit of the backbone of both polysaccharides that are linked by β-1,4 glycosidic units [6]. The only structural difference is the nitrogen-containing side groups. From a chemical point of view, chitosan is the deacetylated product of chitin. It is interesting to note that the applications of the two molecules are also quite similar, including drug delivery carrier, external application of drug and food preservatives due to their perfect biocompatibility, outstanding antibacterial and gelling properties [7,8,9].

Mechanical property is essential for the performance of biological materials, whether as tissue structure of fungi and animals or in biomedical applications [10]. During recent years, the mechanical properties of chitin and chitosan materials have been studied at the macro level [11]. For example, the concentration, ionic strength, molecular weight and acetylation degree of chitosan can obviously influence the viscoelasticity of chitosan hydrogel [12,13,14,15]. Notably, the mechanism that the molecular parameters influence on the mechanical behaviors including stiffness, elasticity and rupture strength of chitosan gel was studied by Sacco et al. [16]. Their findings suggest that the frequency of glucosamine and *N*-acetyl-glucosamine of chitosan contribute to a subtle structure–property relationship in chitosan gels. Chitin was always used as condensed materials such as film and powder [17]. Chitin nanofiber with perfect mechanical properties was prepared by Ifuku et al. via surface deacetylation, and for the Young’s moduli, tensile strengths were decreased significantly while the fracture strain was effectively improved due to the plasticizing effect [18]. Additionally, the densification, deformation and compaction of chitin and chitosan film materials were studied via several macroscopic mechanical tests [19,20,21]. The behaviors of water-soluble polymers in diluted solutions are important to understand their macro properties and the design of new water-based materials [22,23]. It has been reported that the mechanical and structural behaviors of both chitin and chitosan films can be greatly influenced by changing the pH value even in a small range [24,25]. A complete understanding on the mechanical behaviors of chitin and chitosan in solutions with different pH is important to recognize their structure and dynamic behaviors under physiology situations. However, few research efforts have focused on the molecular properties of chitin and chitosan in diluted solution with variable pH.

Atomic force microscope (AFM) based single-molecule force spectroscopy (SMFS) has shown impressive abilities in investigating the intra/intermolecular interactions of natural or synthetic polymers at the single-molecule level [22,23,26,27,28,29,30,31,32,33,34,35,36,37,38,39,40,41,42,43]. Especially, the mechanical behaviors of single cellulose and amylose in aqueous environments were systematically studied by Cui et al., which provides a paradigm for the analysis of intramolecular and intermolecular interactions of polysaccharides with a similar backbone [32,35,39]. Recently, the relationship between the structural features and mechanical behaviors of chitin and chitosan in DI water were investigated by Qian et al. via SMFS [42,43]. The authors claim that the deacetylation from chitin to chitosan will increase the number of binding water molecules (solubility) around the chain, further increasing the binding water energy of a single chain. Previous single-molecule studies on chitin or chitosan are concerned more with their mechanical behaviors in a pure solvent. In order to understand their interaction process in physiological conditions, it is necessary to further explore the single-molecule behaviors of chitin and chitosan in more complex aqueous conditions (for example, under different pH).

In this study, SMFS studies were performed in different diluted solutions to investigate the elastic behaviors of chitin and chitosan in solutions with different pH. The SMFS results indicate that compared to in DI water, the existence of H^+^ in acid conditions can increase the single-molecule elasticity of both chitin and chitosan by increasing the number of water bridges located between adjacent structural units. However, the tendency for OH^−^ in alkali to influence molecular elasticity of the two polysaccharides is opposite. It is believed that the molecule mechanical behavior of the two polysaccharides is mainly determined by the composition of interchain and intrachain hydrogen bonds influenced by the side groups. This study may be referential for the design of intelligent pH-responsive biomaterials.

## 2. Materials and Methods

### 2.1. Materials

Chitin and chitosan powder were produced by Aladdin Corp. (Shanghai, China). V-shaped Si_3_N_4_ AFM cantilevers (SNL-10) were purchased from Bruker Corp. (Billerica, MA, USA). The ultrapure water is deionized (DI) (>18 MΩ·cm). DMSO, trimethylsiloxane, NaOH and HCl (37%) were produced by Sigma-Aldrich Corp. (St. Louis, MO, USA).

### 2.2. Details of AFM-Based SMFS

Chitosan was dissolved in DI water (stirred at 23 °C for 5 h) and then diluted to 10 mg/L. The turbid fluid of chitin in DI water was centrifuged at high speed (5000 rpm) and then the supernatant was used as the sample solution in SMFS. The concentration of chitin (about 0.7 ppm) was measured via an inductively coupled plasma-mass (NexlON 1000G, Perkin Elmer, Walsham, MA, USA) spectrometer. Although the “insolubility” of chitin in water covered up information about its solution properties, 0.7 ppm is sufficient for SMFS studies [44]. A quartz slide was preliminarily cleaned ultrasonically for 10 min with H_2_O. Then, the slide was further treated by plasma clean in vacuum for 5 min. After that, the slide was immersed into the mixed solution of concentrated sulfuric acid (98%) and hydrogen peroxide (30%) at a volume ratio of 7:3 for 2 h at 90 °C. One should be careful because the mixed solution has strong corrosiveness. Through these steps, the substrate with the hydroxylated surface can be obtained, which can be used in SMFS experiments, in most cases. Considering that the poor solubility of chitin in DI water may decrease the possibility to capture a single-chitin chain in SMFS experiments, it is necessary to enrich the micro-nano structure of the substrate surface in order to adsorb the molecular chains better. Therefore, trimethylsiloxane was used as the surface coupling agent. About 20 μL of trimethylsiloxane was dropped in a brown bottle with 10 mL of ethyl alcohol to form a homogenous solution. Then, the hydroxylated slide was immersed independently in the bottle and sealed for 1 h. It should be pointed out that since trimethylsiloxane has very high reactivity, the whole process must avoid light and water to inhibit the cross-linking reaction on the solution and surface. After that, the surface-silanized quartz slide was taken out and ultrasonicated three times with tetrahydrofuran, ethanol and deionized water, respectively. Then, the indicated liquid of the slide was dried with compressed nitrogen. Finally, the slide was dried at 80 °C for 30 min and used for SMFS experiments immediately after the surface restored to room temperature.

Before SMFS experiments, about 15 μL of the polysaccharide solution was dropped on a clean quartz slide for 15–30 min. The sample was then flushed by the liquid used in the SMFS experiments (Cypher VRS, Asylum Research, Santa Barbara, CA, USA). The parameters of the SMFS experiments were set according to the pre-experimental results. In general, the magnitude of the force applied by the AFM probe on the substrate surface can greatly affect the probability of obtaining a single-molecule stretching event. The larger loading force will increase the interaction between the AFM probe and the substrate, and the molecular bridge based on the force-induced covalent interaction is likely to be formed between the probe and the substrate. In this way, single-molecule stretching events with higher force values can be obtained. However, this way may also lead to an increasing adhesion effect between the AFM probe and the substrate, and cover up the stretching information of the molecular chain. The final loading force in this study was set as 4 nN. Another important experimental parameter in single-molecule experiments is the adsorption time of the sample solution on the substrate surface. A long adsorption time may lead to entanglement and stacking between molecular chains so that single-chain molecules cannot be pulled out during SMFS. If the adsorption time is too short, the molecular chain is likely to be unstable, resulting in a lower stretching force. In this study, the adsorption time for chitin and chitosan is 30 min and 15 min, respectively. The choice of the AFM probe is also important for SMFS experiments. The ability of different types of probes to capture molecules and the environmental noise that they generate are also differentiated. In order to capture single-stranded polysaccharide molecules as much as possible, we used a probe with the V-shaped Si_3_N_4_ AFM cantilever and a large radius of curvature (Bruker Corp., Billerica, MA, USA). The movement rate of the probe should be controlled in an appropriate range to ensure that the stretching of the molecular chain is in an equilibrium or quasi-equilibrium state. The movement rate of the AFM tip is 2.0 μm/s in this study, which is a typical value for SMFS. The residence time of the probe on the substrate surface and furthest from the substrate surface are both set as 0.5 s, which can increase the molecular capture efficiency. The spring constant of the AFM cantilever (about 40 pN/nm) was calibrated by the thermal noise method.

During the experiment, the cantilever approached the sample surface to pick up the polymer chain, then retracted gradually away from the surface to stretch the chain. Meanwhile, the values of the piezo movement and the force exerted on the cantilever were recorded by the instrument. The signal was converted into force-extension (F-E) curves, subsequently. All F-E curves were further analyzed with Igro Pro (Wavemetrics, Portland, OR, USA) and Python scripts. In order to prevent the influence of volatilization on the test results, only the first 100 data were taken for each single-molecule force spectrum experiment under acidic and alkaline conditions, and the test of each sample was completed within 15 min. The single-molecule force spectrum experiment was carried out in a closed environment. Before and after the SMFS experiments, the AFM probe and the quartz optical lens were cleaned with alcohol and deionized water, respectively, to avoid cross-interference caused by different samples and environments. In order to ensure the reliability of the experimental results, SMFS experiments were performed on 5 samples under each experimental condition, and the valid data obtained under each condition is not less than 20.

## 3. Results and Discussion

### 3.1. The Inherent Elastic Behaviors of Chitin and Chitosan

**Figure 2:**
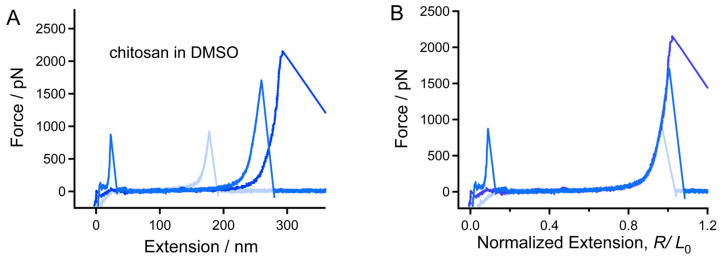
Typical F-E curves of chitosan obtained in DMSO. (**A**) The original F-E curves. (**B**) The normalized effects of those shown in (**A**).

In order to compare the mechanical behavior of the backbone of chitin and chitosan, force tests were performed in a strong H-bond destroyer (DMSO) [32,35,42,45,46,47]. Figure 1 shows the typical force-extension (F-E) curve of single-chitosan chains in DMSO. The force peak which appears at the initial part corresponds to the adhesive force between the AFM tip and quartz surface [44]. The maximum force and the length value of the adhesive peaks of different stretching events vary because of the existence of micro-nano structure on the surface. Subsequently, the resilience force of the chain gradually increases from 0 to the maximum value, and then drops to 0 speedily when the molecular bridge breaks [36]. Therefore, an outstanding force peak appears at the termination of the F-E curve, which corresponds to the elastic elongation of the molecule. Because the molecular weight of the molecules varies, the contour length measured by SMFS differs from each other, which can be reflected by the stretching distance from 0 nm to extension at the apex of the of the last force peak of the curves (Figure 2A).

**Figure 3:**
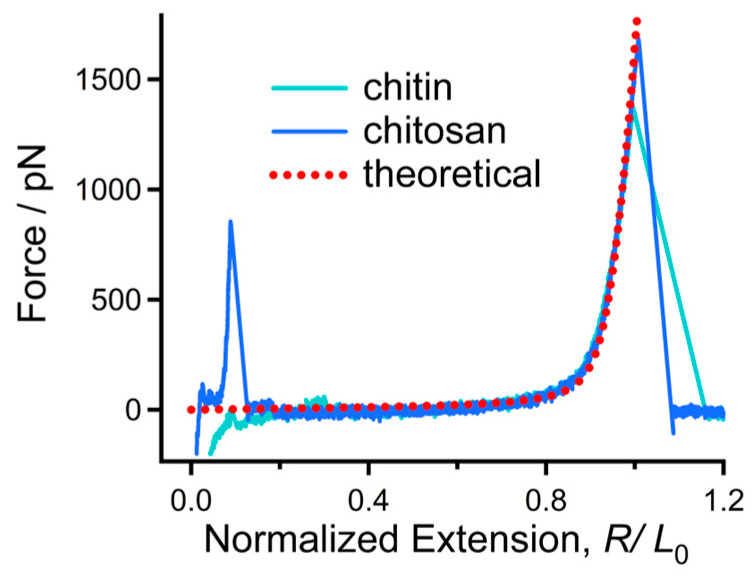
Experimental F-E of chitin and chitosan compared with the theoretical curve from QM-FJC model.

It has been reported that molecules that share the same chemical structure show a similar single-chain elasticity in an organic solvent, in most cases [37,48]. In order to compare the elasticity of chitosan molecules with different contour length, the F-E curves were normalized at a high force (1500 pN in this study). One can note that the normalized F-E curves of chitosan (Figure 2B) can be superposed nicely in the whole force-extension region. This result indicates that these curves correspond to the elastic elongation of single-chitosan chains. The F-E curves of chitin are shown in Figure S1, which can also be superposed well with each other. Typical normalized F-E curves of chitin and chitosan obtained in the environment (DMSO) are compared together for further analysis (Figure 3). It is obvious that the curves are superposed well in the elastic elongation force region, indicating that chitin and chitosan share the similar molecular mechanical behavior in DMSO. Because the molecular elasticity of a polymer chain is determined by the covalent and noncovalent interactions along the chain direction [30], we suggest that the intrachain weak interactions of both chitin and chitosan may be destroyed in DMSO. Therefore, the molecules show their inherent elasticity that is determined by the β-1,4 glycosidic backbone.

The theoretical elastic modulus of β-1,4 glycosidic backbone was calculated by Cui et al. via quantum mechanical (QM) calculations on single-cellulose chains by including the theoretical elastic modulus into a wormlike chain (FJC) model. The modified model (QM-FJC model, Equation (1), can be used to describe the elastic behaviors of β-1,4 glycosidic backbone [39].

*R*/*L*_0_ = (*L*[*F*]/*L*_0_){coth[(*Fl_K_*])/(*k_B_T*)] − (*k_B_T*)/(*Fl_K_*)} (1)

In Equation (1), *R/L*_0_ corresponds to the extension after normalization of a polymer, *L*[*F*] is the chain length at F, *L*_0_ is the chain length at free state, *k_B_* is the Boltzmann constant, *T* is the Kelvin temperature and *l_K_* is the Kuhn length of the polymer chain. It should be pointed out that the value of *l_K_* is closely related to the flexibility or rigidity of a polymer chain; the larger *l_K_* corresponds to a more rigid chain.

As shown in Figure 3, the theoretical F-E curve of the β-1,4 glycosidic backbone can be superposed with the experimental F-E curve of chitin and chitosan in DMSO when *l_K_* = 0.514 nm (the average length of the repeating unit of chitin and chitosan). This result manifests that both chitin and chitosan show their inherent elasticity in DMSO, indicating that the nonbonding effects along the chain direction can be ignored in this case. This result may be beyond expectations because both chitin and chitosan possess a large number of H-bond donors. We suggest the result should be attributed to the competition between intrachain and intermolecular H-bonds [35]. DMSO can break intrachain H-bonds along the chain direction, and the number of intermolecular H-bonds between DMSO and chitin/chitosan increases accordingly. Because DMSO has only H-bond acceptors, it is hard to form a molecular bridge along a chitin or chitosan chain via H-bond effect [48]. When the chain is elongated, the force contribution from the strength of intermolecular H-bonds is negligible [47]. Therefore, both chitin and chitosan show the inherent elasticity of β-1,4 glycosidic backbone in DMSO

### 3.2. The Elastic Behaviors of Chitin and Chitosan in DI Water and Acid Conditions

**Figure 4:**
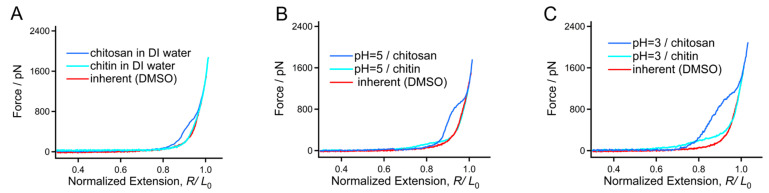
F-E curves of chitin and chitosan obtained in DI water (**A**), pH = 5 (**B**) and pH = 3 (**C**) compared with the inherent F-E curve.

We further studied the single-chain mechanical behaviors of these two biomacromolecules in aqueous conditions since water is a key biological metabolite and solvent [32]. As shown in Figure 4A, the typical F-E curve of chitin obtained in DI water can be superposed with the typical F-E curve obtained in DMSO in most of the force region, but shows slight deviation in the force region from 200–500 pN. As a comparison, the deviation between the F-E curves of chitosan obtained in DMSO and DI water is much apparent (from 0–800 pN). All the F-E curves can be superposed well at the high forces (higher than 500 pN for chitin and 800 pN for chitosan, respectively), where it is governed by the configuration of the backbone. It is interesting to note that the force deviation between the F-E curves obtained in DI water and DMSO for chitosan is much larger than that for chitin, indicating that the noncovalent bonding energy along a chitosan chain is larger than chitin. It has been widely reported that the amino group can be ionized through binding H^+^ in solution [49]. H_3_O^+^ on the side groups may further form by binding water molecules. It is interesting that the H-bond donors and acceptor on H_3_O^+^ are helpful to the formation of a “water bridge” between the adjacent structural units of the polymer. Moreover, the charge repulsion between the adjacent H_3_O^+^ may prompt that the chain exists as an extended conformation; then, more water molecules bind the chain through H-bonds [48]. Therefore, the larger force deviation between the F-E curves obtained in DI water and DMSO for chitosan comparing to chitin may be mainly attributed to the more exposed H-bond donors and acceptors of the side groups.

Furthermore, force measurements were performed in acid conditions to study the influence of H^+^ on the two molecules. For both chitin and chitosan, the force deviation between the F-E curves obtained in acid solution (pH = 5) and DMSO is quite larger than that between DI water and DMSO (Figure 4B), indicating that H^+^ can increase the number of water bridges for both chitin and chitosan. Moreover, the deviation for chitosan is much larger than that of chitin in the two conditions (0–250 pN for chitin and 0–1000 pN for chitosan). The result is consistent with the conclusion from microscopic studies that the deformation quantity and viscoelasticity of both chitin and chitosan nanofibers, and chitosan shows a more acid sensitivity [50,51,52]. In a solution with a higher H^+^ concentration (pH = 3), the deviation tendency is more obvious (0–500 pN for chitin and 0–1500 pN for chitosan, Figure 4C). A more obvious deviation corresponds to a larger noncovalent interaction along the chain direction. Therefore, we can make a conclusion that a higher H^+^ concentration may be helpful to increase the single-molecule elasticity of both chitin and chitosan. Because the number of water bridges for chitosan is more sensitive to pH value than chitin, its mechanical variation is also more outstanding than chitin in the same range of pH values. The SMFS result can also explain the fact that the solubility of chitosan can be obviously improved in acid conditions.

### 3.3. The Mechanical and Thermodynamic Properties of Chitin and Chitosan Determined by pH Value

**Figure 5:**
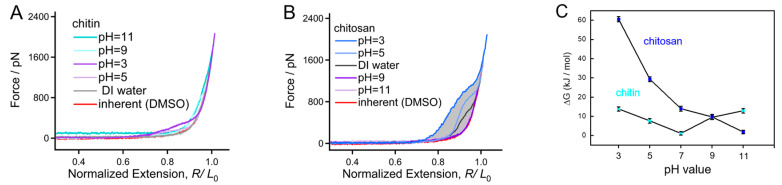
Comparison of chitin (**A**) and chitosan (**B**) obtained in aqueous conditions with different pH. (**C**) The nonbonding energy along the chain of chitin and chitosan.

Alkalinity can obviously increase the solubility of typical water insoluble polysaccharides (such as cellulose and chitin) [53]. SMFS experiments of chitin and chitosan were performed in alkali solutions with pH gradient to investigate the internal mechanism that OH^−^ effects on a chitin/chitosan molecule. As shown in Figure 5A, comparing DI water and acid conditions, the typical F-E curves of chitin obtained in alkali solutions are much higher at the high forces, causing a force deviation if the force is larger than 400 pN. In addition, the force deviation in this region increases with the concentration of OH^−^. Notice that the F-E curve obtained under high OH^−^ concentration (pH = 11) appears to be a stable force plateau (about 80 pN), which is consumed to overcome the hydrophobic interaction when the chain is stretched from a collapsed state [37]. It should be pointed out that although both H^+^ and OH^−^ can cause the force deviation for chitin in the low force region (below 500 pN), the influence from OH^−^ is much obvious at the high forces (below 1600 pN). The force deviation for a polar molecule at low and high force regions is mainly influenced by intramolecular and intermolecular interactions, respectively. Therefore, it can be speculated that chitin suffers stronger intrachain interaction in aqueous alkali, which may be attributed to its collapsed compact conformation. It seems that this conclusion conflicts with the fact that OH^−^ increases the solubility of chitin in water. However, a synergistic effect from temperature and other components (such as urea and salts) must be considered when discussing the solubility of chitin [54]. For a chitosan molecule, the force deviation between the F-E curves obtained in aqueous conditions compared to that obtained in DMSO decreases with the increasing pH value (Figure 5B). This result manifests that the molecular elasticity of chitosan can be enhanced by H^+^ while weakened by OH^−^ in aqueous conditions.

The mechanical behaviors of chitin and chitosan in alkaline conditions are quite different. The noncovalent bonding energy along chain direction (ΔG) of chitin and chitosan in aqueous solutions can be obtained by calculating the area of the force deviation between the F-E curves obtained in an aqueous solution and DMSO. In order to have a comprehensive understanding, the ΔG of chitin and chitosan along the chain direction in various pH conditions are compared directly (Figure 5C). One can see clearly that the ΔG of chitosan shows a negative correlation with the pH value in the entire experimental pH region, and the ΔG decreases from a large value (about 60 kJ/mol) to about 0 kJ/mol when the pH increases from 3 to 11. For chitin, however, even both H^+^ and OH^−^ can increase the ΔG; the variation of ΔG is not outstanding (not more than 15 kJ/mol). The huge difference in pH sensitivity for chitin and chitosan at the single-molecule level is mainly determined by the side groups, which is essential to hydration. The acetyl group of chitin is hydrophobic compared to the amino group of chitosan, leading to a weak hydration and doughy pH sensitivity. On the contrary, the strong polarity of the amino group makes chitosan a perfect hydration and more sensitive to the structural change of the water network induced by pH variation. The ΔG values of chitin and chitosan in different aqueous conditions perfectly explains the fact that chitin is always used in powder materials while the application of chitosan in aqueous conditions is frequent.

## 4. Conclusions

In brief, the mechanical and thermodynamics properties of chitin and chitosan in solutions are studied through SMFS at the single-molecule level. The results manifest that the two molecules share the same inherent single-molecule mechanics because they possess the same backbone, and the influence from the difference of side groups can be ignored. However, the single-molecule behaviors of chitin and chitosan show huge difference in aqueous conditions. Especially, the noncovalent bonding energy of chitosan along the chain direction can be increased up to a about 60 kJ/mol in acid condition, which is almost the highest environment-induced noncovalent bonding energy increase known. Due to the existence of a relatively hydrophobic acetyl group, the variation of noncovalent bonding energy value for chitin in aqueous conditions with different pH is much lower (within 15 kJ/mol) than that of chitosan. This study can not only be helpful to understand the macroscopic mechanics and structural behaviors of chitin and chitosan under variable pH conditions, but also inspire the development of chitin and chitosan-based pH responsive smart biohydrogel with considerable elasticity.

## Data Availability

Data are contained within the article and Supplementary Materials.

## Supplementary Materials

The following supporting information can be downloaded at: https://www.mdpi.com/article/10.3390/polym16070995/s1, Figure S1: The typical F-E curves of chitin obtained in DMSO; Figure S2: The normalized F-E curves of chitosan obtained in DI water; Figure S3: The normalized F-E curves of chitosan obtained under pH = 9; Figure S4: The normalized F-E curves of chitosan obtained under pH = 11; Figure S5: The normalized F-E curves of chitosan obtained under pH = 5; Figure S6: The normalized F-E curves of chitosan obtained under pH = 3; Figure S7: The normalized F-E curves of chitin obtained under pH = 9; Figure S8: The normalized F-E curves of chitin obtained under pH = 11; Figure S9: The nor-malized F-E curves of chitin obtained under pH = 5; Figure S10: The normalized F-E curves of chitin obtained under pH = 3; Table S1: The theoretical elastic modulus of cellulose backbone calculated by Cui et al. [55].
