# Supplementary material for: Effect of Environmental pH on the Mechanics of Chitin and Chitosan: A Single-Molecule Study
Source: Polymers (Basel). 2024 Apr 5;16(7):995. doi: 10.3390/polym16070995 (PMC11014069; doi:10.3390/polym16070995)
Supplement: Supplementary file 1 [file polymers-16-00995-s001.zip › polymers-2856448-supplementary.pdf]

# Supporting Information

# Effect of Environmental pH on the Mechanics of Chitin and Chitosan: A Single-Molecule Study

**Song Zhang *, Yunxu Ji, Yiwei He, Juan Dong, Haohang Li and Shirui Yu ***

Department of Food Science and Engineering, Moutai Institute, Renhuai 564502, China; jiyunxu@mtxy.edu.cn (Y.J.); 18185657179@139.com (Y.H.); 18586724865@163.com (J.D.); lyz20180810lyz@163.com (H.L.)

* Correspondence: zhangsong1@mtxy.edu.cn (S.Z.); yushirui@mtxy.edu.cn (S.Y.)

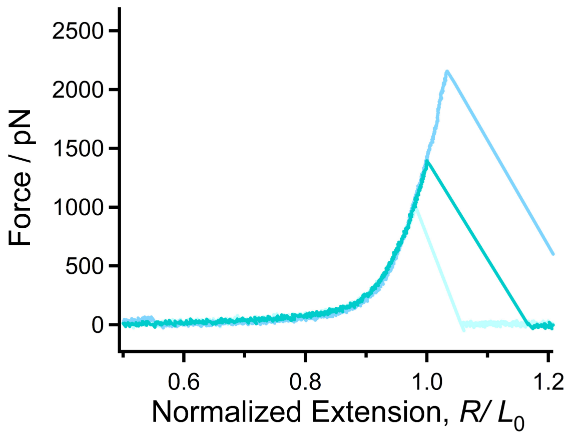


Figure S1 The typical F-E curves of chitin obtained in DMSO

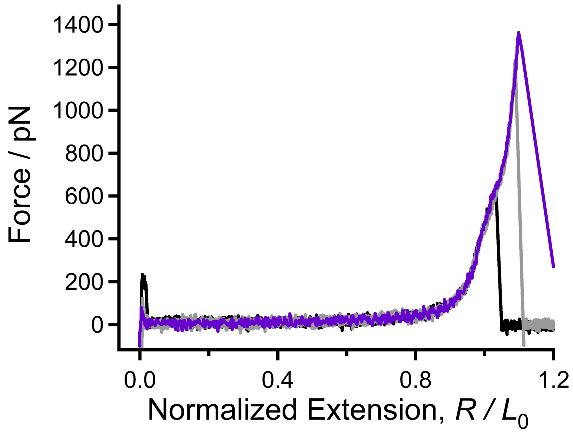


Figure S2 The normalized F-E curves of chitosan obtained in DI water

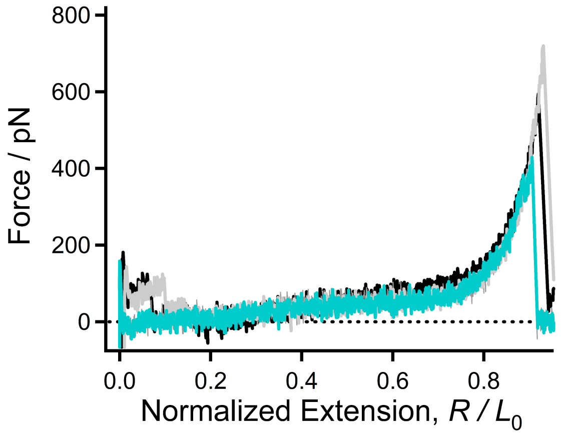


Figure S3 The normalized F-E curves of chitosan obtained under pH=9

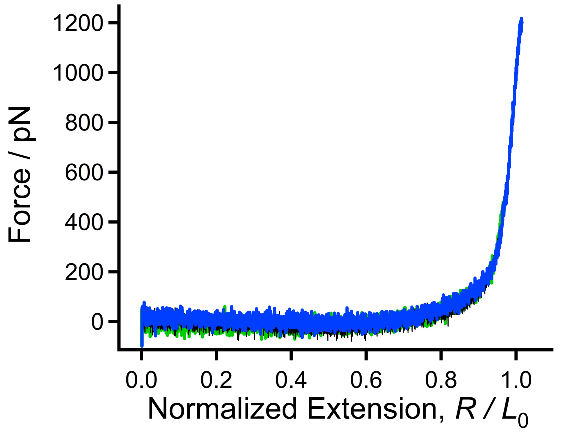


Figure S4 The normalized F-E curves of chitosan obtained under pH=11

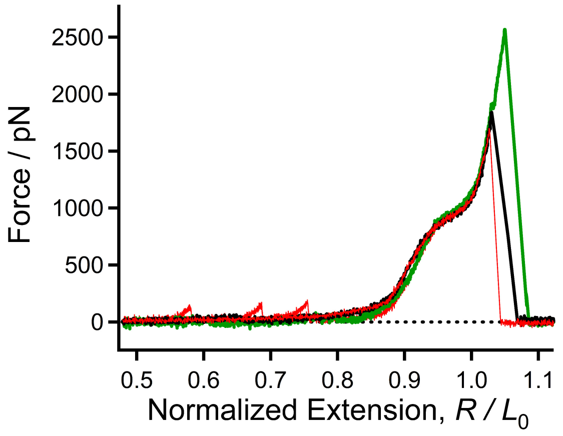


Figure S5 The normalized F-E curves of chitosan obtained under pH=5

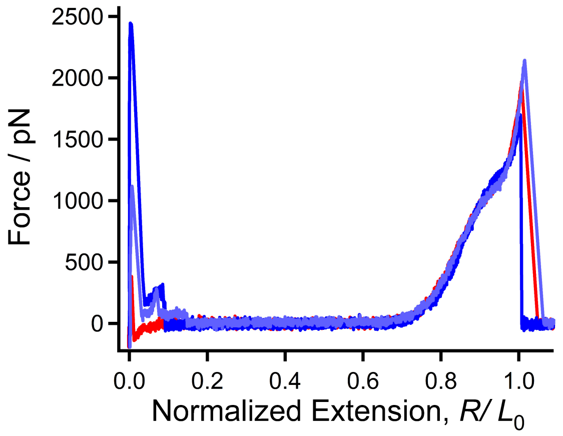


Figure S6 The normalized F-E curves of chitosan obtained under pH=3

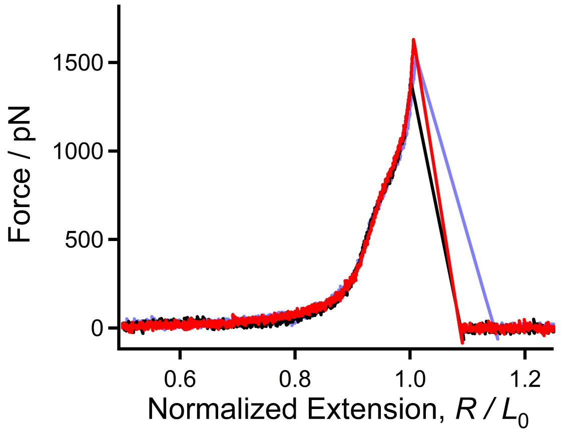


Figure S7 The normalized F-E curves of chitin obtained under pH= 9

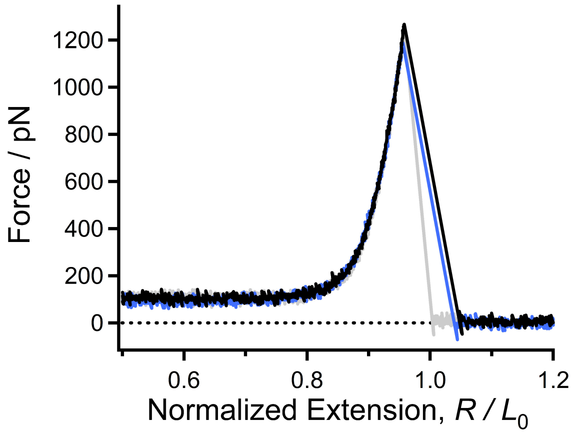


Figure S8 The normalized F-E curves of chitin obtained under pH= 11

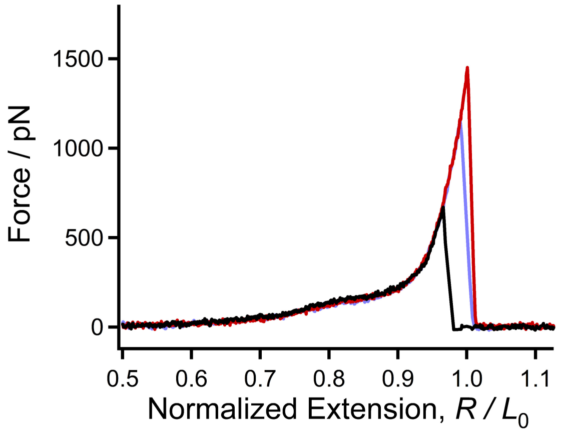


Figure S9 The normalized F-E curves of chitin obtained under pH= 5

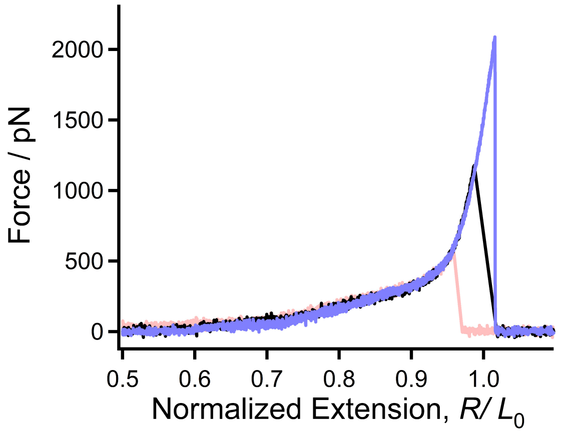


Figure S10 The normalized F-E curves of chitin obtained under pH= 3

Table S1 The theoretical elastic modulus of cellulose backbone calculated by Cui et al. [1].

| γ1 | γ2 | γ3 |
|---|---|---|
| 10.57 nN | 90.23 nN | -44.12 nN |

Supporting References


1. Bao, Y.; Qian, H. J.; Lu, Z. Y.; Cui, S. Revealing the Hydrophobicity of Natural Cellulose by Single-Molecule Experiments. Macromolecules 2015, 48, 3685-3690.
